# Expression of CCCTC‐binding factor (CTCF) is linked to poor prognosis in prostate cancer

**DOI:** 10.1002/1878-0261.12597

**Published:** 2019-11-29

**Authors:** Doris Höflmayer, Amélie Steinhoff, Claudia Hube‐Magg, Martina Kluth, Ronald Simon, Eike Burandt, Maria Christina Tsourlakis, Sarah Minner, Guido Sauter, Franziska Büscheck, Waldemar Wilczak, Stefan Steurer, Hartwig Huland, Markus Graefen, Alexander Haese, Hans Heinzer, Thorsten Schlomm, Frank Jacobsen, Andrea Hinsch, Alexandra M. Poos, Marcus Oswald, Karsten Rippe, Rainer König, Cornelia Schroeder

**Affiliations:** ^1^ Institute of Pathology University Medical Center Hamburg‐Eppendorf Germany; ^2^ Martini‐Clinic Prostate Cancer Center University Medical Center Hamburg‐Eppendorf Germany; ^3^ Department of Urology Charité ‐ Universitätsmedizin Berlin Germany; ^4^ Integrated Research and Treatment Center Center for Sepsis Control and Care (CSCC) Jena University Hospital Germany; ^5^ Network Modeling Leibniz Institute for Natural Product Research and Infection Biology ‐ Hans Knöll Institute Jena Germany; ^6^ Faculty of Biosciences Heidelberg University Germany; ^7^ Division of Chromatin Networks German Cancer Research Center (DKFZ) and Bioquant Heidelberg Germany; ^8^ General, Visceral and Thoracic Surgery Department and Clinic University Medical Center Hamburg‐Eppendorf Germany

**Keywords:** CTCF, deletion, prostate cancer, TMA

## Abstract

The chromatin‐organizing factor CCCTC‐binding factor (CTCF) is involved in transcriptional regulation, DNA‐loop formation, and telomere maintenance. To evaluate the clinical impact of CTCF in prostate cancer, we analyzed CTCF expression by immunohistochemistry on a tissue microarray containing 17 747 prostate cancers. Normal prostate tissue showed negative to low CTCF expression, while in prostate cancers, CTCF expression was seen in 7726 of our 12 555 (61.5%) tumors and was considered low in 44.6% and high in 17% of cancers. Particularly, high CTCF expression was significantly associated with the presence of the transmembrane protease, serine 2:ETS‐related gene fusion: Only 10% of ERG‐negative cancers, but 30% of ERG‐positive cancers had high‐level CTCF expression (*P* < 0.0001). CTCF expression was significantly associated with advanced pathological tumor stage, high Gleason grade (*P* < 0.0001 each), nodal metastasis (*P* = 0.0122), and early biochemical recurrence (*P* < 0.0001). Multivariable modeling revealed that the prognostic impact of CTCF was independent from established presurgical parameters such as clinical stage and Gleason grade of the biopsy. Comparison with key molecular alterations showed strong associations with the expression of the Ki‐67 proliferation marker and presence of phosphatase and tensin homolog deletions (*P* < 0.0001 each). The results of our study identify CTCF expression as a candidate biomarker for prognosis assessment in prostate cancer.

## Graphical abstract

The transcriptional repressor CCCTC‐binding factor, which is involved in modeling the chromatin face in the nucleus of a cell, was analyzed for its expression in prostate cancer. We report here that its upregulation was associated with a shorter prostate‐specific antigen recurrence‐free survival after prostatectomy in a large cohort of prostate cancer patients.

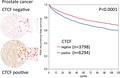

**Abbreviations**
CTCF: CCCTC‐binding factor
FISH: fluorescence *in situ* hybridization
IHC: immunohistochemistry
Ki67‐LI: Ki67 labeling index
PSA: prostate‐specific antigen
*PTEN*: phosphatase and tensin homolog
TMA: tissue microarray
*TMPRSS2:ERG*: transmembrane protease, serine 2:ETS‐related gene fusion

## 1 Introduction

Prostate cancer has an incidence of 76 in 100 000 men and a mortality rate of 10 in 100 000 men in Western Europe (Bray *et al.*, 2018). This means that one out of seven prostate cancer patient has a rather aggressive disease. In localized disease, the decision between radical prostatectomy and observation is guided by established pretreatment prognostic parameters [Gleason grade and tumor extent on biopsies, preoperative prostate‐specific antigen (PSA), and clinical stage] (Thompson and Tangen, 2012; Wilt *et al.*, 2012). In retrospective studies, these parameters are statistically powerful. For individual treatment decisions, their specificity, sensitivity, and predictive value are suboptimal. Thus, it is hoped that new clinically applicable molecular markers will enable a more reliable prediction of prostate cancer aggressiveness.

CCCTC‐binding factor (CTCF) is a ubiquitously expressed transcription factor characterized by 11 zinc fingers binding to more than 20 000 DNA loci in the human genome (Ohlsson *et al.*, 2001). By mediating inter‐ and intrachromosomal interactions, CTCF is essential for the three‐dimensional chromatin organization (Ong and Corces, 2014) and participates in the regulation of DNA methylation and transcriptional activity (Guastafierro *et al.*, 2008). In addition, CTCF is involved in the regulation of telomerase as reviewed previously in a complex manner (Ramlee *et al.*, 2016). The binding of CTCF to the first exon of the hTERT gene was reported to suppress its expression in telomerase‐negative cells but not in cancer cells in a DNA methylation‐dependent manner (Renaud *et al.*, 2007). In line with this finding, treatment with a histone deacetylase inhibitor induced histone hyperacetylation and loss of CpG methylation, which facilitated CTCF binding at this locus (Meeran *et al.*, 2010). Furthermore, CTCF was reported to be involved in the spatial organization of the subtelomeres and linked to regulating hTERT expression via binding to an upstream enhancer (Eldholm *et al.*, 2014) as well as transcription of the telomeric TERRA transcript and stability of the shelterin complex (Deng *et al.*, 2012). Thus, it is not surprising that deregulation of CTCF has been observed in many human cancer types. For example, overexpression of CTCF has been reported to occur in breast cancer (Docquier *et al.*, 2005), cervical cancer (Velazquez‐Hernandez *et al.*, 2015), ovarian cancer (Zhao *et al.*, 2017), and hepatocellular carcinoma (Zhang *et al.*, 2017), and has been linked to adverse tumor features in some of them (Zhang *et al.*, 2017; Zhao *et al.*, 2017). Little is known about the clinical impact of CTCF expression in prostate cancer. However, genome‐wide association studies revealed single nucleotide polymorphisms in the CTCF region associated with prostate cancer risk and functional studies in cell lines demonstrated an impact of CTCF knockdown on prostate cell proliferation, migration, and invasion (Chen *et al.*, 2015; Shan *et al.*, 2019). Such data suggest a biologically relevant role of CTCF in prostate cancer (Whitington *et al.*, 2016).

To learn more on the impact of CTCF expression on the clinical course of prostate cancer, we took advantage of our large tissue microarray (TMA) resource including more than 17 000 prostate cancers. The database attached to our TMA contains pathological and clinical follow‐up data, as well as abundant molecular data on key molecular alterations of this disease such as ERG fusion and various genomic deletions.

## 2 Materials and methods

### 2.1 Patients

The 17 747 patients had radical prostatectomy between 1992 and 2014 at the Department of Urology and the Martini Clinics at the University Medical Center Hamburg‐Eppendorf. The entire prostate was embedded and analyzed with a standard procedure (Schlomm *et al.*, 2008). Classical Gleason grading was done along with ‘quantitative’ Gleason grading reflecting the percentage of Gleason 4 patterns as described before (Sauter *et al.*, 2016). Follow‐up was available for 14 464 patients (median 48 months, range 1–275 months; Table S1). PSA recurrence was defined as a postoperative PSA level of ≥ 0.2 ng·mL^−1^ or increasing levels in subsequent measurements. The TMA was produced with 0.6 mm spots as described earlier in detail (Kononen *et al.*, 1998; Mirlacher and Simon, 2010). Each TMA contained various control tissues and normal prostate tissue. The TMA was annotated with results on ERG expression (Minner *et al.*, 2011), ERG break‐apart fluorescence *in situ* hybridization (FISH) (Tsourlakis *et al.*, 2016), Ki67 labeling index (Ki67‐LI) (Tennstedt *et al.*, 2012), and deletion status of 5q21 (CHD1 (Burkhardt *et al.*, 2013), 6q15 (MAP3K7) (Kluth *et al.*, 2013), 10q23 [phosphatase and tensin homolog (*PTEN*)] (Krohn *et al.*, 2012), and 3p13 (FOXP1 (Krohn *et al.*, 2013). Archived diagnostic leftover tissues were pseudo‐anonymized and used without consent in accordance with the local law (HmbKHG, §12a) and approved by the local ethics committee (Ärztekammer Hamburg, WF‐049/09). The work has been carried out in compliance with the Helsinki Declaration.

### 2.2 Immunohistochemistry (IHC)

Freshly cut TMA sections were stained on the same day and experiment. Slides were dewaxed and exposed to heat‐induced antigen retrieval for 5 min at 121 °C in pH 7.8 Tris/EDTA/citrate buffer. The anti‐CTCF polyclonal rabbit antibody HPA004122 Sigma (Merck, Darmstadt, Germany) was applied at 37 °C for 60 min at 1 : 150 dilution (Uhlen *et al.*, 2015). Bound antibody was then visualized using the EnVision Kit (Dako, Glostrup, Denmark) according to the manufacturer's directions. CTCF staining was validated with positive and negative control tissues and found in the nucleus of positive cells. Complete absence of staining was scored as ‘negative’, and a ‘low’ score was given to cancers with a staining intensity of 1+, or 2+ in ≤ 70% of tumor cells, or 3+ in ≤ 30% of tumor cells. The score was ‘high’ if staining intensity was 2+ in > 70% of tumor cells or 3+ in > 30% of tumor cells.

### 2.3 Statistics

Contingency tables were calculated to study association, and the chi‐square test was used to find significant relationship between CTCF expression and clinicopathological variables. Analysis of variance and *F*‐test were applied to find association between CTCF expression and Ki67‐LI. Kaplan–Meier analysis and log‐rank test were applied to test differences in PSA recurrence after prostatectomy. Cox proportional hazards regression analysis was performed to test independence and significance of pathological, molecular, and clinical variables. jmp 12 (SAS Institute Inc., Cary, NC, USA) was used.

## 3 Results

### 3.1 CTCF staining

**Figure 1:**
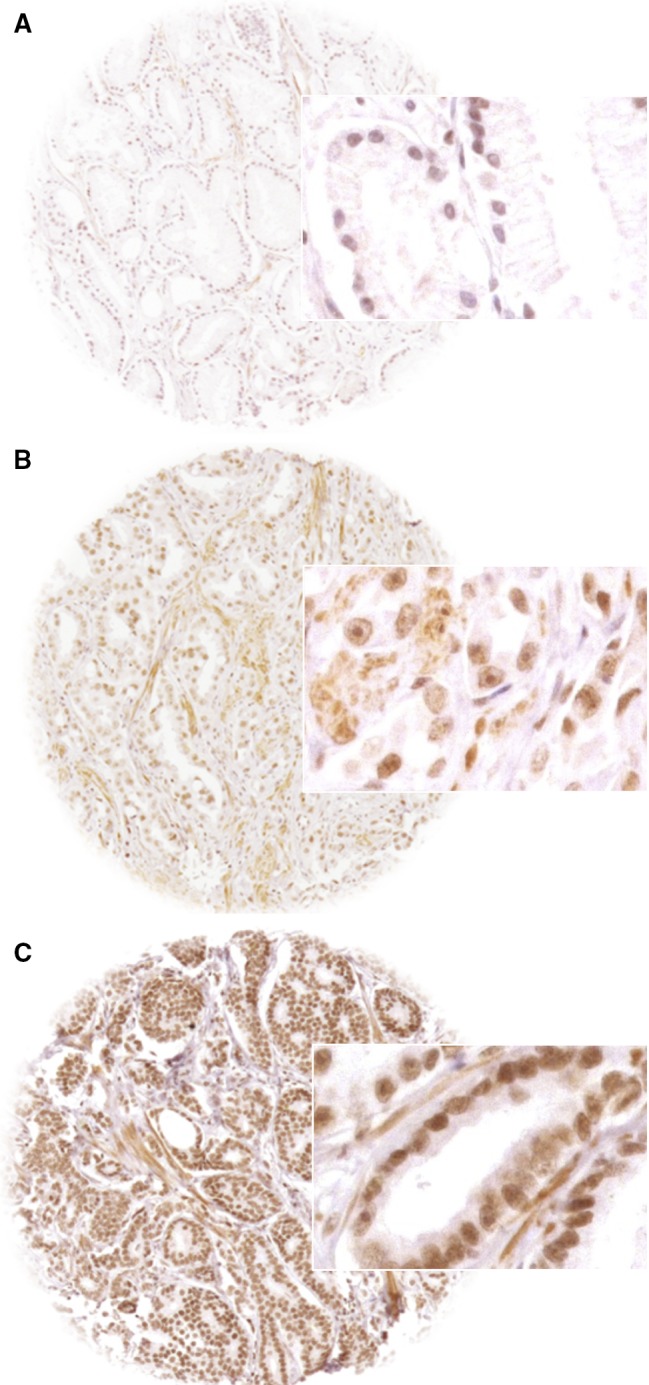
Representative pictures of (A) negative, (B) low, and (C) high CTCF staining in prostate cancer. Spot size is 600 µm at 100/400× original.

A total of 12 555 (71%) tumor samples were interpretable. The remaining 5192 spots (29%) were noninformative because the tissue sample lacked completely or had no unequivocal cancer cells. At the selected 1 : 150 dilution of the anti‐CTCF antibody HPA004122, normal prostate tissue showed negative to low nuclear CTCF expression in basal and luminal cells. In cancers, detectable nuclear CTCF staining was seen in 7726 of our 12 555 (61.5%) tumors and was considered low in 44.6% and high in 17% of cancers. Representative images of CTCF staining are given in Fig. 1.

### 3.2 Association with *TMPRSS2:ERG* fusion status and ERG protein expression

**Figure 2:**
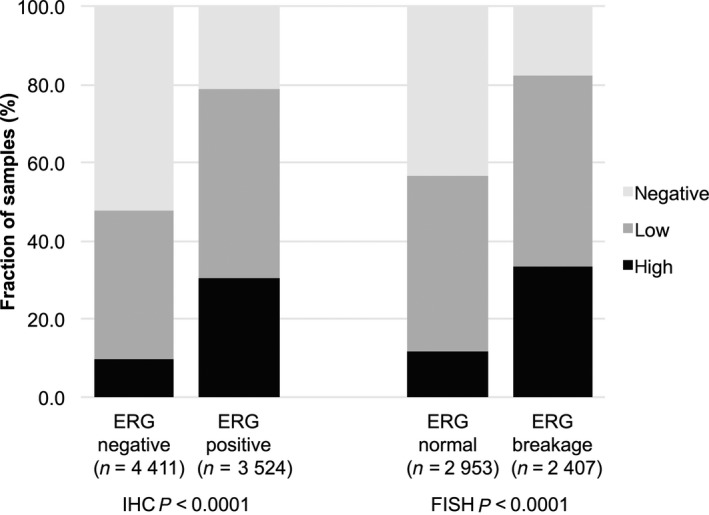
Association between CTCF staining and ERG status (IHC/FISH).

Data on ERG expression obtained by IHC and on transmembrane protease, serine 2:ETS‐related gene fusion (*TMPRSS2:ERG*) rearrangement obtained by FISH were available from 7935 and from 5360 cancers with interpretable CTCF staining results. Data on both ERG FISH and IHC were available from 5191 cancers, and an identical result (ERG IHC positive and break by FISH or ERG IHC negative and missing break by FISH) was found in 4970 of 5191 (96%) cancers. CTCF staining was strongly linked to *TMPRSS2:ERG* rearrangement and ERG positivity in our set of prostate cancers (*P* < 0.0001, Fig. 2).

### 3.3 Association with tumor phenotype

**Figure 3:**
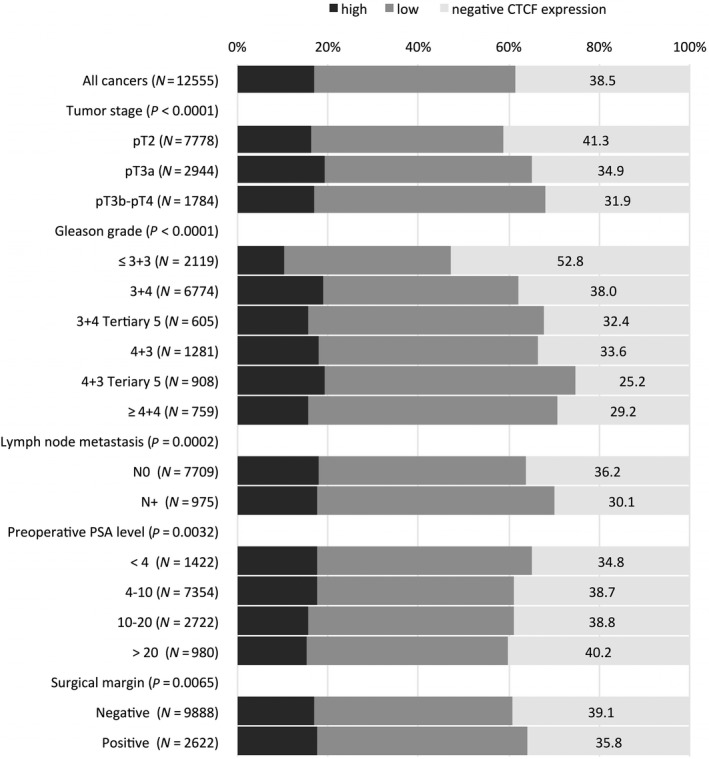
Association between CTCF staining and prostate cancer phenotype.

**Figure 4:**
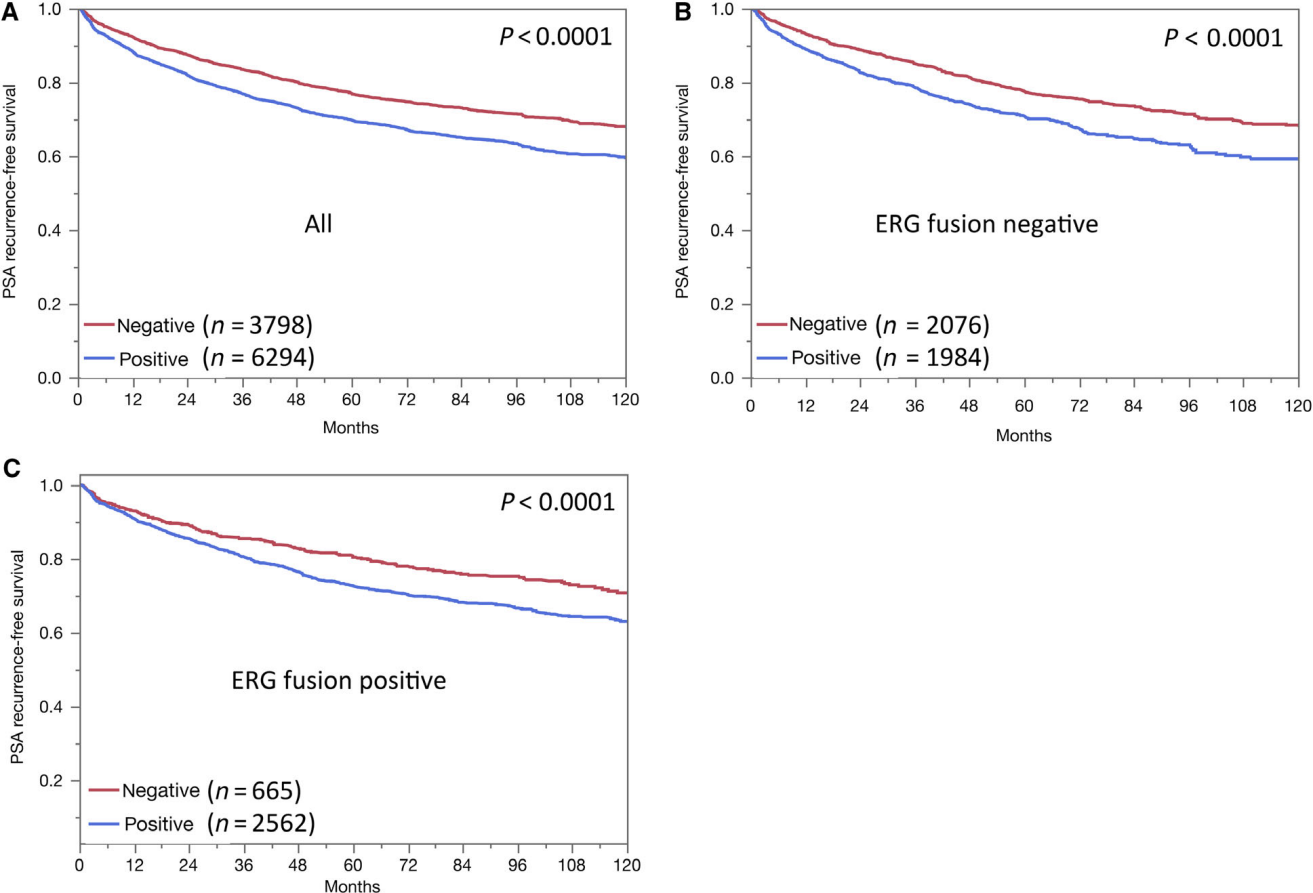
Association between negative and positive CTCF expression and biochemical recurrence in (A) all cancers, (B) the ERG‐negative, and (C) the ERG‐positive subset.

CCCTC‐binding factor staining was significantly linked to advanced tumor stage, high Gleason grade, and presence of lymph node metastasis (*P* ≤ 0.0002 each, Fig. 3). The increase in the percentage of CTCF expression for stage and lymph node metastasis was restricted to the low CTCF group and not seen in the high group, indicating that grouping in CTCF negative vs. positive as shown in Fig. 4 is the more appropriate model for the prognostic effect of CTCF in prostate cancer (Tables S2 and S3).

### 3.4 Association with PSA recurrence

Positive CTCF staining was significantly associated with early PSA recurrence. Subset analyses revealed that this association was true for both ERG‐negative and ERG‐positive cancers (*P* < 0.0001, Fig. 4). To better understand the prognostic power of CTCF, we performed further subset analyses in cancers with identical classical and quantitative Gleason scores. Here, CTCF staining did not provide clear‐cut prognostic information beyond the Gleason score, neither in any subsets defined by the classical Gleason score (Fig. S1a) nor by the quantitative Gleason score (Fig. S1b–h).

### 3.5 Association with genomic deletions and tumor cell proliferation

**Figure 5:**
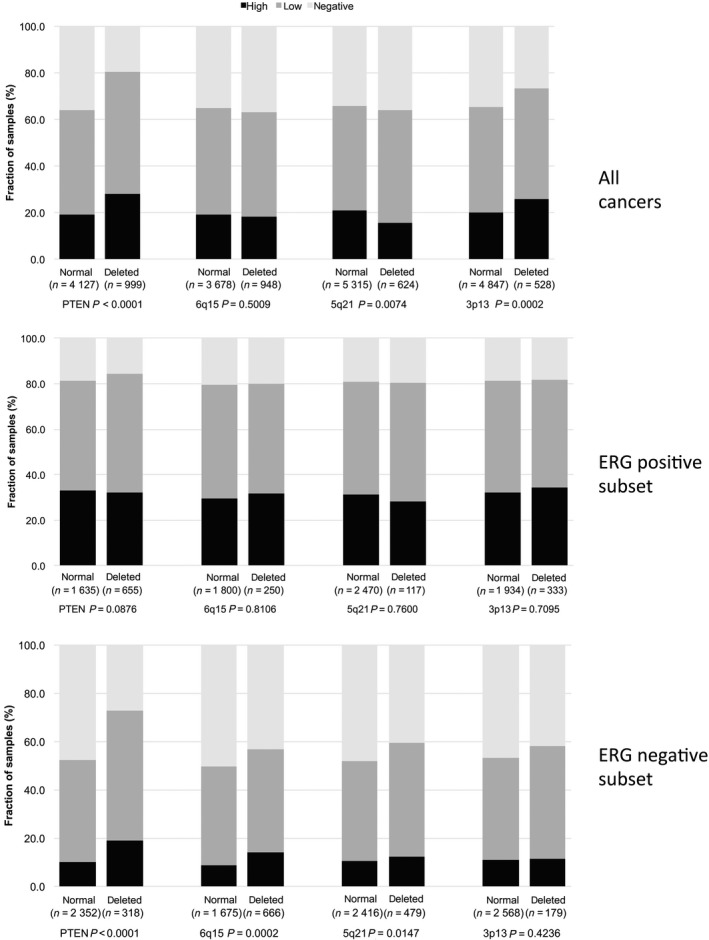
Association between CTCF staining and 10q23 (*PTEN*), 5q21 (CHD1), 6q15 (MAP3K7), 3p13 (FOXP1) deletions in all cancers, the ERG‐negative, and the ERG‐positive subset.

CCCTC‐binding factor staining was strongly associated with *PTEN* deletions when all cancers were jointly analyzed (*P* < 0.0001, Fig. 5). This held also true in the subset of ERG‐negative cancers (*P* < 0.0001), while this association was lost in ERG‐positive cancers (*P* = 0.0876). CTCF expression was significantly linked to increased cell proliferation as measured by Ki67‐LI (Table 1). The average Ki67‐LI increased from 2.1 ± 0.05 in cancers lacking CTCF expression to 3.20 ± 0.06 in cancers with low and to 3.42 ± 0.09 in cancers with high CTCF levels (*P* < 0.0001). This association held true in all tumor subsets with identical Gleason score (≤ 3 + 3: *P* < 0.0001, 3 + 4: *P* < 0.0001, 4 + 3: *P* = 0.006, ≥ 4 + 4: *P* = 0.0205).

**Table 1:** Association between CTCF staining results and Ki67‐LI in various Gleason categories. SEM, standard error of the mean.

Gleason	CTCF expression	*N*	Ki67‐LI mean	±SEM	*P*
Total	Negative	2333	2.09	0.05	< 0.0001
	Low	2229	3.20	0.06	
	High	930	3.42	0.09	
≤ 3 + 3	Negative	622	1.84	0.08	< 0.0001
	Low	408	2.64	0.10	
	High	102	2.72	0.21	
3 + 4	Negative	1248	1.96	0.06	< 0.0001
	Low	1228	3.01	0.07	
	High	611	3.22	0.09	
3 + 4 Tertiary 5	Negative	85	2.61	0.29	0.0024
	Low	100	3.48	0.27	
	High	35	4.46	0.46	
4 + 3	Negative	210	2.68	0.23	0.006
	Low	235	3.56	0.21	
	High	90	3.74	0.35	
4 + 3 Tertiary 5	Negative	89	2.52	0.39	0.0006
	Low	141	4.45	0.31	
	High	48	4.00	0.54	
≥ 4 + 4	Negative	79	3.43	0.52	0.0205
	Low	115	4.75	0.43	
	High	42	5.81	0.71	

### 3.6 Multivariable analyses

Multivariable analyses were performed in all cancers and the subset of ERG‐negative and ERG‐positive cancers evaluating the clinical relevance of CTCF expression in different scenarios (Table 2, Table S4). The results of these analyses demonstrated a weak independent prognostic role of CTCF in the preoperative setting with a hazard ratio of 1.30 and a *P*‐value < 0.0001.

**Table 2:** Cox proportional hazards for PSA recurrence‐free survival after prostatectomy of established preoperative prognostic parameter and CTCF expression.

Variable		*N*	Univariable analysis	Multivariable analysis (*N* = 8624)
Gleason grade biopsy	≥ 4 + 4 vs. ≤ 3 + 3	12 172	5.91 (5.33–6.55)***	4.01 (3.54–4.54)***
cT‐stage	T3a vs. T1c	14 404	2.15 (1.72–2.65)***	1.46 (1.11–1.88)*
Preoperative PSA level	≥ 20 vs. <4	14 611	5.06 (4.41–5.81)***	3.44 (2.84–4.17)***
CTCF expression	Positive vs. negative	10 227	1.37 (1.26–1.48)***	1.30 (1.19–1.42)***
ERG‐negative subset	Positive vs. negative	4120	1.40 (1.24–1.58)***	1.27 (1.12–1.43)** ^,^ a
ERG‐positive subset	Positive vs. negative	3297	1.40 (1.18–1.67)***	1.44 (1.20–1.72)*** ^,^ b

Confidence interval (95%) in brackets; asterisk indicates significance level:

a *N* = 4014.

b *N* = 3182.

* *P* ≤ 0.05

** *P* ≤ 0.001

*** *P* ≤ 0.0001.

## 4 Discussion

The results of our study show that CTCF expression is linked to poor outcome in prostate cancer.

Our immunohistochemical analysis revealed detectable CTCF staining in 61.5% of 12 555 analyzable prostate cancers. The level of immunostaining was typically higher in cancers than in normal prostate glands, the latter of which showed mostly negative and only sometimes weak CTCF expression. This suggests that CTCF becomes upregulated during tumor development and/or progression in a relevant fraction of prostate cancers. Similar findings have been reported from other solid cancer types. For example, CTCF was more strongly expressed in breast and cervical cancers as compared to very low CTCF levels detected in normal breast tissues and in low‐grade intraepithelial lesions of the cervix (Docquier *et al.*, 2005; Velazquez‐Hernandez *et al.*, 2015).

The significant association of elevated CTCF expression with unfavorable tumor phenotype, including high Gleason grade, advanced tumor stage, presence of lymph node metastasis (*P* ≤ 0.0002, each), accelerated cell proliferation (*P* < 0.0001), and early biochemical recurrence (*P* ≤ 0.001), argues for a role of CTCF for prostate cancer progression and aggressiveness. This view is supported by recent data from Shan et al. showing that prostate cancer cell line growth (LNCap, PC‐3) in nude mice is promoted by CTCF (Shan *et al.*, 2019). A tumor‐promoting role of CTCF is also supported by studies in other cancer types. For example, CTCF upregulation was linked to advanced or metastatic disease and poor prognosis in hepatocellular carcinoma and ovarian cancers (Zhang *et al.*, 2017; Zhao *et al.*, 2017). CTCF has a multifunctional role enabling chromatin looping for interactions between distal enhancers and proximal promoters. It is likely that altered CTCF expression can cause deregulation of many genes. In fact, it has been shown that many tumor‐related genes become affected by CTCF expression changes, including p53, retinoblastoma protein, c‐myc, insulin‐like growth factor 2, p14, p16, and Fox0 (Fiorentino and Giordano, 2012; Shan *et al.*, 2019). Interestingly, structural DNA changes induced by CTCF have also been implicated with telomere maintenance and tumor cell immortality as CTCF is supposed to prevent telomere DNA damage signaling (Deng *et al.*, 2012; Renaud *et al.*, 2007).

The highly annotated TMA allowed us to further study CTCF upregulation in molecularly defined subgroups. About 50% of all prostate cancers carry a gene fusion linking the androgen‐regulated serine protease TMPRSS2 with the ETS‐transcription factor ERG resulting in an androgen‐related overexpression of ERG (Brase *et al.*, 2011; Tomlins *et al.*, 2005; Weischenfeldt *et al.*, 2013). The intriguing association between strong CTCF expression and ERG fusion is compatible with the role of CTCF for the development of genomic rearrangements (Canela *et al.*, 2017). It is well known that CTCF increases the risk for translocations through induction of chromosomal proximity (Handoko *et al.*, 2011; Ong and Corces, 2014) and that it is even implicated in DNA looping involving ETS genes (Qin *et al.*, 2015; Taslim *et al.*, 2012). Comparison with recurrent genomic deletions identified *PTEN* as the only deletion that was linked to strong CTCF expression. *PTEN* deletion is the main reason for hyperactive PI3K/AKT signaling in prostate cancer and is associated with tumor growth, progression, and poor clinical outcome (Taslim *et al.*, 2012). An effect of *PTEN* inactivation on CTCF expression fits well with earlier reports linking *PTEN* to CTCF via MYC: Loss of *PTEN* triggers MYC upregulation (Kaur and Cole, 2013), which is an upstream activator of CTCF expression (Klenova *et al.*, 2002).

The results of our multivariable modeling identify CTCF as a candidate marker that could help to guide therapy decisions at the stage of the needle biopsy. However, it is of note that the Gleason grade is the strongest (and least expensive) prognostic feature in prostate cancer. In a recent analysis, we have demonstrated that by using the percentage of unfavorable Gleason patterns, the Gleason grading can be transformed from a categorical into a continuous variable with an even finer distinction of prognostic subgroups (Sauter *et al.*, 2018; Sauter *et al.*, 2016). That CTCF lacks prognostic impact in cancers with identical (classical and quantitative) Gleason is another proof for the unprecedented prognostic power of Gleason scoring when it is performed in a specialized center.

The multifunctional role of CTCF in cancer biology may open new avenues for novel targeted anticancer therapies. For example, it has been shown that CTCF knockdown causes an anti‐apoptotic effect in breast cancer cells (Docquier *et al.*, 2005). Furthermore, CTCF can regulate TERT expression and induce telomere instability (Ramlee *et al.*, 2016; Renaud *et al.*, 2007). It is, thus, tempting to speculate that prostate cancer patients with CTCF expression may benefit from novel therapies targeting telomere instability once they become available. For example, putative telomere‐associated target structures may include the telomeric G‐strand, components of the telomere synthesis machinery, or telomere protection proteins such as shelterin, the molecular target of gemcitabine (Fadri‐Moskwik *et al.*, 2013).

## 5 Conclusions

In summary, our study shows that CTCF expression is a prognostic unfavorable feature in prostate cancer, but CTCF is a candidate biomarker with low predictive power.

## Conflict of interest

The authors declare no conflict of interest.

## Author contributions

DH, CS, RS, AS, and GS designed the study and drafted the manuscript. HHu, MG, AH, HHe, RK, and KR participated in the study design. EB, CS, MCT, and SM performed IHC analysis and scoring. FB, FJ, WW, and SS participated in pathology data analysis. CH, CS, and RS performed statistical analysis. AH, TS, MK, AMP, and MO participated in data interpretation and helped to draft the manuscript. All authors read and approved the final manuscript.

## Supporting information


**Table S1** **.** Pathological and clinical data of the arrayed prostate cancers.
**Table S2** **.** Association between CTCF staining results and prostate cancer phenotype in the ERG negative subset.
**Table S3** **.** Association between CTCF staining results and prostate cancer phenotype in the ERG fusion positive subset.
**Table S4** **.** Multivariable analysis including CTCF expression in all cancers, the ERG negative and the ERG positive subset.
**Fig. S1** **.** Prognostic impact of CTCF expression in subsets of cancers defined by a) the classical Gleason score categories and b–h) the quantitative Gleason score categories defined by the percentage of b) ≤ 5%, c) 6–10%, d) 11–20%, e) 21–30%, f) 31–49%, g) 50–60%, and h) 61–100% Gleason 4 patterns.
