# Supplementary material for: Expression of CCCTC‐binding factor (CTCF) is linked to poor prognosis in prostate cancer
Source: Mol Oncol. 2019 Nov 29;14(1):129–38. doi: 10.1002/1878-0261.12597 (PMC6944131; doi:10.1002/1878-0261.12597)
Supplement: Supplementary file 1 — Table S1 . Pathological and clinical data of the arrayed prostate cancers. Table S2 . Association between CTCF staining results and prostate cancer phenotype in the ERG negative subset. Table S3 . Association between CTCF staining results and prostate cancer phenotype in the ERG fusion positive subset. Table S4 . Multivariable analysis including CTCF expression in all cancers, the ERG negative and the ERG positive subset. Fig. S1 . Prognostic impact of CTCF expression in subsets of cancers defined by a) the classical Gleason score categories and b–h) the quantitative Gleason score categories defined by the percentage of b) ≤ 5%, c) 6–10%, d) 11–20%, e) 21–30%, f) 31–49%, g) 50–60%, and h) 61–100% Gleason 4 patterns. [file MOL2-14-129-s001.docx]

**Table S1.** Pathological and clinical data of the arrayed prostate cancers

|  | **No. of patients (%)** | |
| --- | --- | --- |
|  | **Study cohort on TMA** | **Biochemical relapse among categories** |
|  | **(N=17,747)** |  |
| **Follow-up** |  |  |
| N | 14,464 | 3,612 (25%) |
| Mean / median (month) | 56.3 / 48.0 | - |
| **Age (y)** |  |  |
| ≤50 | 433 | 66 (15.2%) |
| 51-59 | 4,341 | 839 (19.3%) |
| 60-69 | 9,977 | 2,073 (20.8%) |
| ≥70 | 2,936 | 634 (21.6%) |
| **Pretreatment PSA (ng/ml)** | |  |
| <4 | 2,225 | 313 (14.1%) |
| 4-10 | 10,520 | 1,696 (16.1%) |
| 10-20 | 3,662 | 1,043 (28.5%) |
| >20 | 1,231 | 545 (44.3%) |
| **pT stage (AJCC 2002)** |  |  |
| pT2 | 11,518 | 1,212 (10.5%) |
| pT3a | 3,842 | 1,121 (29.2%) |
| pT3b | 2,233 | 1,213 (54.3%) |
| pT4 | 85 | 63 (74.1%) |
| **Gleason grade** |  |  |
| ≤3+3 | 3,570 | 264 (7.4%) |
| 3+4 | 9,336 | 1,436 (15.4%) |
| 3+4 Tertiary 5 | 1,697 | 165 (9.7%) |
| 4+3 | 2,903 | 683 (23.5%) |
| 4+3 Tertiary 5 | 1,187 | 487 (41%) |
| ≥4+4 | 999 | 531 (53.2%) |
| **pN stage** |  |  |
| pN0 | 10,636 | 2,243 (21.1%) |
| pN+ | 1,255 | 700 (55.8%) |
| **Surgical margin** |  |  |
| Negative | 14,297 | 2,307 |
| Positive | 3,388 | 1,304 |

NOTE: Numbers do not always add up to 17,747 in the different categories because of cases with missing data. Abbreviation: AJCC, American Joint Committee on Cancer.

**Table S2.** Association between CTCF staining results and prostate cancer phenotype in the ERG *negative* subset

|  | **CTCF** | | | |  |  |
| --- | --- | --- | --- | --- | --- | --- |
| **Parameter** | **N** | **Negative (%)** | **Low (%)** | **High (%)** |  | **P** |
| **ERG negative subset** | 4,411 | 52.1 | 38.0 | 9.9 |  |  |
|  |  |  |  |  |  |  |
| **Tumor stage** |  |  |  |  |  | <0.0001 |
| pT2 | 2,887 | 55.2 | 36.1 | 8.7 |  |  |
| pT3a | 931 | 49.6 | 38.5 | 11.9 |  |  |
| pT3b-pT4 | 584 | 40.2 | 46.9 | 12.8 |  |  |
|  |  |  |  |  |  |  |
| **Gleason grade** |  |  |  |  |  | <0.0001 |
| ≤3+3 | 807 | 69.0 | 26.5 | 4.5 |  |  |
| 3+4 | 2,371 | 52.0 | 37.7 | 10.2 |  |  |
| 3+4 Tertiary 5 | 197 | 44.2 | 46.2 | 9.6 |  |  |
| 4+3 | 498 | 45.8 | 41.8 | 12.4 |  |  |
| 4+3 Tertiary 5 | 277 | 36.5 | 48.0 | 15.5 |  |  |
| ≥4+4 | 259 | 34.7 | 52.5 | 12.7 |  |  |
|  |  |  |  |  |  |  |
| **Lymph node metastasis** |  |  |  |  |  | 0.0122 |
| N0 | 2,596 | 48.5 | 40.1 | 11.5 |  |  |
| N+ | 262 | 38.9 | 46.9 | 14.1 |  |  |
|  |  |  |  |  |  |  |
| **Preoperative PSA level (ng/ml)** |  |  |  |  |  | 0.0311 |
| <4 | 444 | 46.4 | 42.1 | 11.5 |  |  |
| 4-10 | 2,621 | 53.1 | 37.2 | 9.8 |  |  |
| 10-20 | 965 | 51.7 | 39.8 | 8.5 |  |  |
| >20 | 362 | 52.2 | 34.8 | 13.0 |  |  |
|  |  |  |  |  |  |  |
| **Surgical margin** |  |  |  |  |  | 0.4605 |
| Negative | 3,488 | 52.5 | 37.7 | 9.8 |  |  |
| Positive | 914 | 50.2 | 39.4 | 10.4 |  |  |

**Table S3.** Association between CTCF staining results and prostate cancer phenotype in the ERG fusion *positive* subset

|  | **CTCF** | | | |  |  |
| --- | --- | --- | --- | --- | --- | --- |
| **Parameter** | **N** | **Negative (%)** | **Low (%)** | **High (%)** |  | **P** |
| **ERG positive subset** | 3,524 | 21.1 | 48.3 | 30.5 |  |  |
|  |  |  |  |  |  |  |
| **Tumor stage** |  |  |  |  |  | 0.2134 |
| pT2 | 2,077 | 21.7 | 47.2 | 31.1 |  |  |
| pT3a | 948 | 19.8 | 48.7 | 31.4 |  |  |
| pT3b-pT4 | 483 | 20.1 | 52.6 | 27.3 |  |  |
|  |  |  |  |  |  |  |
| **Gleason grade** |  |  |  |  |  | <0.0001 |
| ≤3+3 | 696 | 32.6 | 50.4 | 17.0 |  |  |
| 3+4 | 2,045 | 19.5 | 45.5 | 35.0 |  |  |
| 3+4 Tertiary 5 | 106 | 15.1 | 55.7 | 29.2 |  |  |
| 4+3 | 341 | 18.2 | 51.6 | 30.2 |  |  |
| 4+3 Tertiary 5 | 192 | 9.4 | 55.2 | 35.4 |  |  |
| ≥4+4 | 141 | 16.3 | 56.0 | 27.7 |  |  |
|  |  |  |  |  |  |  |
| **Lymph node metastasis** |  |  |  |  |  | 0.4435 |
| N0 | 2,019 | 19.5 | 48.1 | 32.4 |  |  |
| N+ | 238 | 16.8 | 52.1 | 31.1 |  |  |
|  |  |  |  |  |  |  |
| **Preoperative PSA level (ng/ml)** |  |  |  |  |  | 0.061 |
| <4 | 474 | 21.1 | 51.7 | 27.2 |  |  |
| 4-10 | 2,150 | 20.0 | 47.6 | 32.4 |  |  |
| 10-20 | 642 | 23.4 | 46.7 | 29.9 |  |  |
| >20 | 229 | 22.7 | 52.4 | 24.9 |  |  |
|  |  |  |  |  |  |  |
| **Surgical margin** |  |  |  |  |  | 0.1389 |
| Negative | 2,743 | 21.6 | 48.3 | 30.1 |  |  |
| Positive | 765 | 18.7 | 48.5 | 32.8 |  |  |

**Table S4.** Multivariable analysis including CTCF expression in all cancers, the ERG negative and the ERG positive subset

| **Tumor  subset** | **Scen-ario*** | **N** | **P value for hazard ratio of PSA recurrence after prostatectomy** | | | | | | | |
| --- | --- | --- | --- | --- | --- | --- | --- | --- | --- | --- |
|  |  |  | **Preoperative PSA-Level** | **pT stage** | **cT stage** | **Gleason grade prostatectomy** | **Gleason grade biopsy** | **pN stage** | **R  margin** | **CTCF-expression** |
|  |  |  |  |  |  |  |  |  |  |  |
| **All cancers** | 1 | 6,636 | <0.0001 | <0.0001 | - | <0.0001 | - | <0.0001 | <0.0001 | 0.0596 |
|  | 2 | 9,959 | <0.0001 | <0.0001 | - | <0.0001 | - | - | <0.0001 | 0.0373 |
|  | 3 | 9,802 | <0.0001 | - | <0.0001 | <0.0001 | - | - | - | 0.0026 |
|  | 4 | 8,488 | <0.0001 | - | <0.0001 | - | <0.0001 | - | - | <0.0001 |
|  |  |  |  |  |  |  |  |  |  |  |
| **ERG negative subset** | 1 | 2,629 | 0.0003 | <0.0001 | - | <0.0001 | - | 0.0007 | 0.2484 | 0.2024 |
|  | 2 | 4,048 | <0.0001 | <0.0001 | - | <0.0001 | - | - | 0.0013 | 0.2050 |
|  | 3 | 4,010 | <0.0001 | - | <0.0001 | <0.0001 | - | - | - | 0.1232 |
|  | 4 | 3,954 | <0.0001 | - | <0.0001 | - | <0.0001 | - | - | 0.0016 |
|  |  |  |  |  |  |  |  |  |  |  |
| **ERG positive subset** | 1 | 2,065 | <0.0001 | <0.0001 | - | <0.0001 | - | 0.0121 | 0.0007 | 0.1958 |
|  | 2 | 3,213 | <0.0001 | <0.0001 | - | <0.0001 | - | - | <0.0001 | 0.0527 |
|  | 3 | 3,161 | <0.0001 | - | <0.0001 | <0.0001 | - | - | - | 0.0326 |
|  | 4 | 3,111 | <0.0001 | - | <0.0001 | - | <0.0001 | - | - | 0.0003 |

* Scenario 4 combines preoperatively available parameters (preoperative PSA, clinical tumor (cT) stage, and Gleason grade obtained on the original biopsy) with the postoperative CTCF expression. In scenario 3 the biopsy Gleason is replaced by the Gleason grade obtained on radical prostatectomy. In scenario 2 cT stage is superseeded by pathological tumor (pT) stage and surgical margin (R) status. In scenario 1 the lymph node (pN) stage is added.





**Fig. S1**. Prognostic impact of CTCF expression in subsets of cancers defined by a) the classical Gleason score categories and b-h) the quantitative Gleason score categories defined by the percentage of b) ≤5%, c) 6-10%, d) 11-20%, e) 21-30%, f) 31-49 %, g) 50-60%, and h) 61-100% Gleason 4 patterns.
